# Longitudinal Anesthetic and Critical Care Management After Emergency Thoracic Endovascular Aortic Repair for Blunt Thoracic Aortic Injury in an Adolescent: A Case Report

**DOI:** 10.7759/cureus.116759

**Published:** 2026-09-22

**Authors:** Vincenzo Cuzzone, Claudia De Stefano, Giuseppe Manna, Giulia Cama, Amelia Paglia

**Affiliations:** 1 Anesthesiology and Critical Care, Ospedale Antonio Cardarelli, Campobasso, ITA

**Keywords:** adolescent, anesthesia, blunt thoracic aortic injury, intensive care, polytrauma, thoracic endovascular aortic repair

## Abstract

Blunt thoracic aortic injury (BTAI) is uncommon in children and adolescents but carries substantial early mortality. Thoracic endovascular aortic repair (TEVAR) can provide rapid definitive control in anatomically suitable patients; however, the subsequent course is often driven by associated injuries and evolving critical illness. A previously healthy 14-year-old boy was transferred after a high-energy road traffic collision with a contained injury of the descending thoracic aorta, mediastinal hematoma, left femoral and open tibial fractures, pulmonary contusions, and splenic contusion. Emergency TEVAR with a 24 × 80-mm thoracic endograft achieved complete exclusion without endoleak. External fixation of the long-bone fractures followed. After initial extubation, the patient required reintubation on hospital day 4 for severe hypoxemia with bilateral pulmonary consolidations and left pleural effusion; he was successfully extubated two days later. On day 21, delayed ileocecal perforation with diffuse peritonitis required emergency right hemicolectomy. Definitive femoral and tibial intramedullary fixation and pleural drainage were performed on day 35, followed by percutaneous drainage of a pelvic abscess on day 44. He was discharged home neurologically intact on day 50, with a patent, well-positioned endograft and no endoleak. This case shows that successful TEVAR is one component of a prolonged, physiology-driven perioperative strategy requiring continuity across vascular, orthopedic, abdominal, respiratory, and rehabilitation care.

## Introduction

Blunt thoracic aortic injury (BTAI) is a highly lethal consequence of rapid deceleration trauma. Among patients who reach the hospital alive, prompt computed tomography angiography (CTA), hemodynamic control, and definitive repair have improved survival [1-3]. The Society for Vascular Surgery (SVS) grading system distinguishes intimal tear (grade I), intramural hematoma (grade II), pseudoaneurysm or contained rupture (grade III), and free rupture (grade IV) [1,4]. Current guidelines favor thoracic endovascular aortic repair (TEVAR) for appropriate higher-grade injuries, while emphasizing individualized timing, associated-injury burden, anatomy, and surveillance [1-3].

Pediatric BTAI remains rare, and device sizing, aortic growth, access-vessel caliber, and lifetime surveillance complicate treatment decisions [5-8]. Published reports commonly emphasize technical success, whereas the anesthetic and critical care trajectory after aortic exclusion may receive less attention. We report the sequential perioperative management of an adolescent with grade III BTAI and severe multisystem trauma whose recovery required repeated reassessment across vascular, orthopedic, abdominal, respiratory, and infectious complications. The report was prepared in accordance with the CARE framework [9].

## Case presentation

Initial assessment and emergency aortic repair

A previously healthy 14-year-old boy was transferred to our trauma center after a high-energy road traffic collision. CTA demonstrated a contained injury immediately distal to the left subclavian artery, with focal external contour abnormality and mediastinal hematoma, consistent with grade III BTAI (Figure 1). Associated injuries included left femoral and open tibial shaft fractures, bilateral pulmonary contusions, and splenic contusion. The patient proceeded directly to emergency repair after multidisciplinary assessment by vascular surgery, anesthesia and intensive care, general surgery, and orthopedics. 

**Figure 1:**
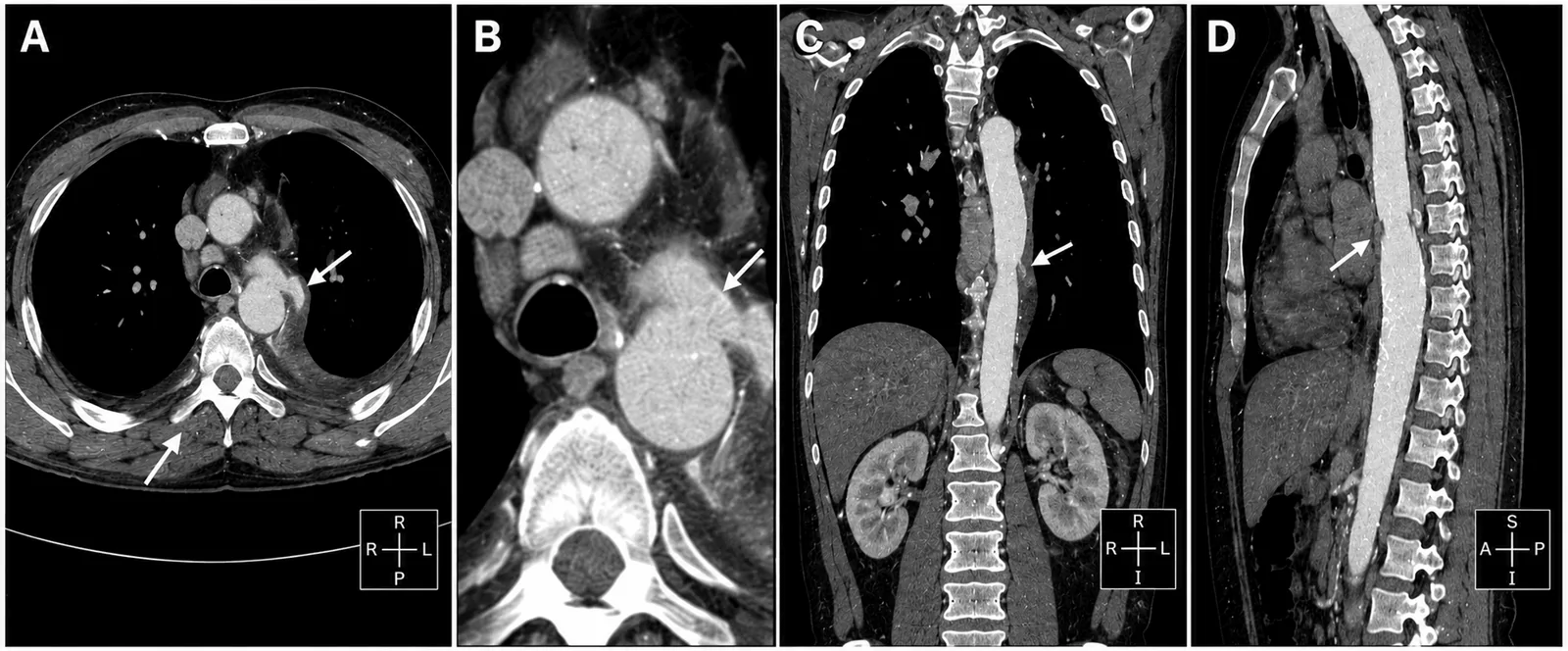
Preoperative computed tomography angiography demonstrating grade III blunt thoracic aortic injury. (A) Axial computed tomography angiography (CTA) shows focal external contour irregularity of the descending thoracic aorta with surrounding mediastinal hematoma. (B) Magnified axial view delineates the focal aortic lesion. (C) Coronal and (D) sagittal CTA reconstructions demonstrate the traumatic aortic injury immediately distal to the left subclavian artery. White arrows indicate the site of the aortic injury.

General anesthesia was conducted with invasive hemodynamic monitoring and avoidance of marked tachycardia, hypertension, and hypotension. Bilateral common femoral arterial exposure was performed. After systemic administration of 5,000 IU unfractionated heparin, a 24 × 80-mm thoracic endograft was deployed below the left subclavian artery. Completion angiography confirmed complete exclusion of the lesion without extravasation or endoleak. Three units of group O Rh-negative packed red blood cells were administered during the emergency operative phase. The patient was transferred intubated to the intensive care unit (ICU). Immediate postoperative CTA confirmed correct endograft position, complete exclusion of the traumatic lesion, and no endoleak (Figure 2).

**Figure 2:**
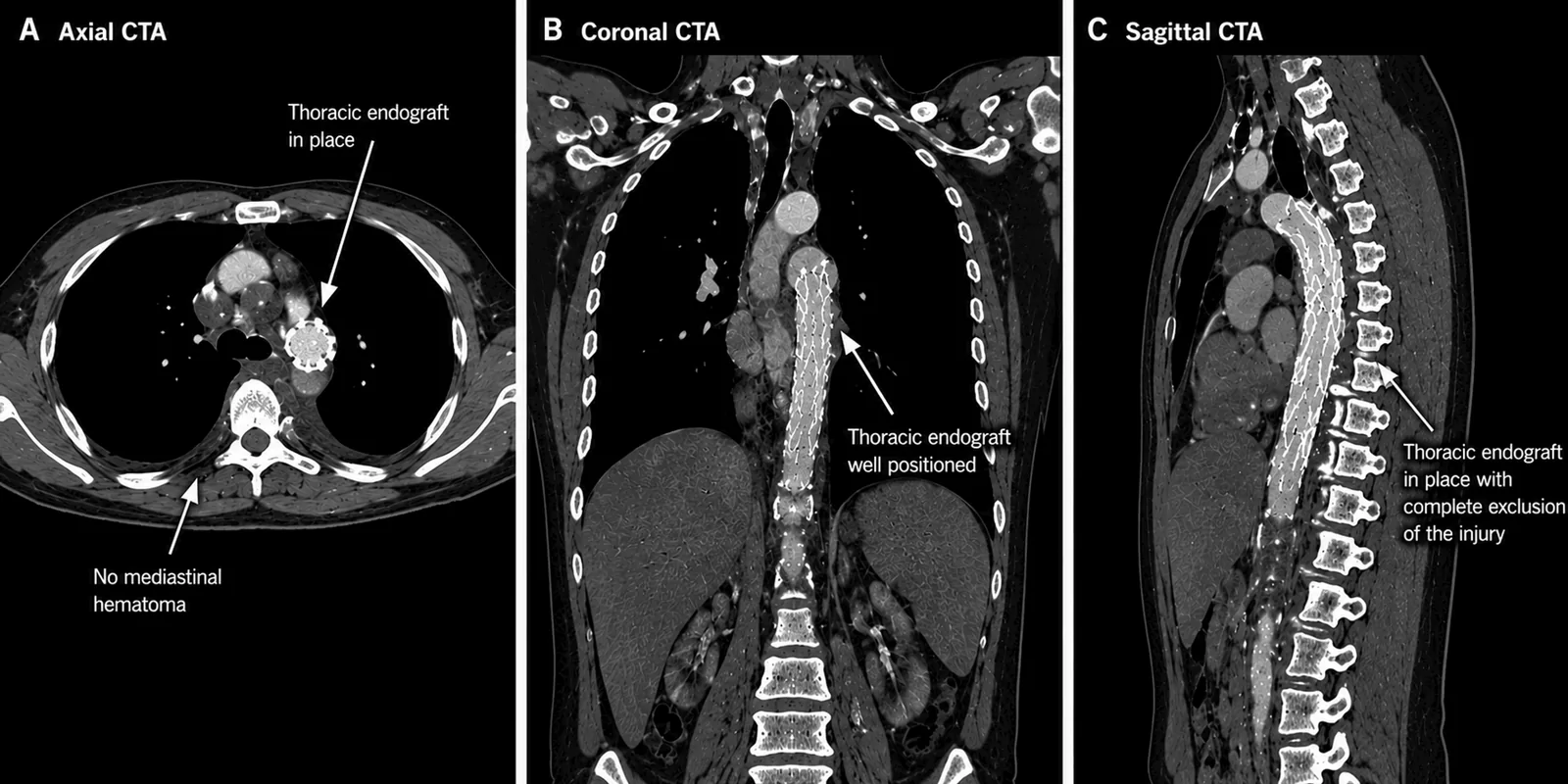
Postoperative computed tomography angiography after thoracic endovascular aortic repair. (A) Axial computed tomography angiography (CTA) demonstrates the thoracic endograft in place and absence of residual mediastinal hematoma. (B) Coronal CTA shows the thoracic endograft correctly positioned along the descending thoracic aorta. (C) Sagittal CTA demonstrates complete exclusion of the traumatic aortic lesion without endoleak. White arrows indicate the thoracic endograft and the resolved mediastinal hematoma, as labeled.

Orthopedic damage control and respiratory deterioration

On hospital day 1, the displaced femoral fracture and open tibial fracture were reduced and stabilized with external fixation under general anesthesia. Lung-protective ventilation, correction of metabolic and electrolyte abnormalities, and conservative fluid titration were used because of the pulmonary contusions. After clinical improvement, the patient was extubated on day 2 and transferred from the ICU.

On day 4, fever, tachycardia, somnolence, and worsening oxygenation developed. Arterial blood gas analysis while receiving 50% oxygen by Venturi mask (Covidien LLC, Mansfield, MA) showed a PaO₂ of 55 mmHg. CTA of the chest demonstrated bilateral pulmonary consolidations and a left pleural effusion (Figure 3). The patient returned to the ICU and underwent tracheal intubation and mechanical ventilation. Empirical antimicrobial therapy was escalated after infectious diseases consultation, and ventilatory, sedative, hemodynamic, and fluid strategies were adjusted to serial gas exchange and clinical findings. A transient episode of bradycardia responded to atropine; echocardiography showed preserved global ventricular function and no pericardial effusion. He was extubated on day 6 after improvement in respiratory mechanics and gas exchange.

**Figure 3:**
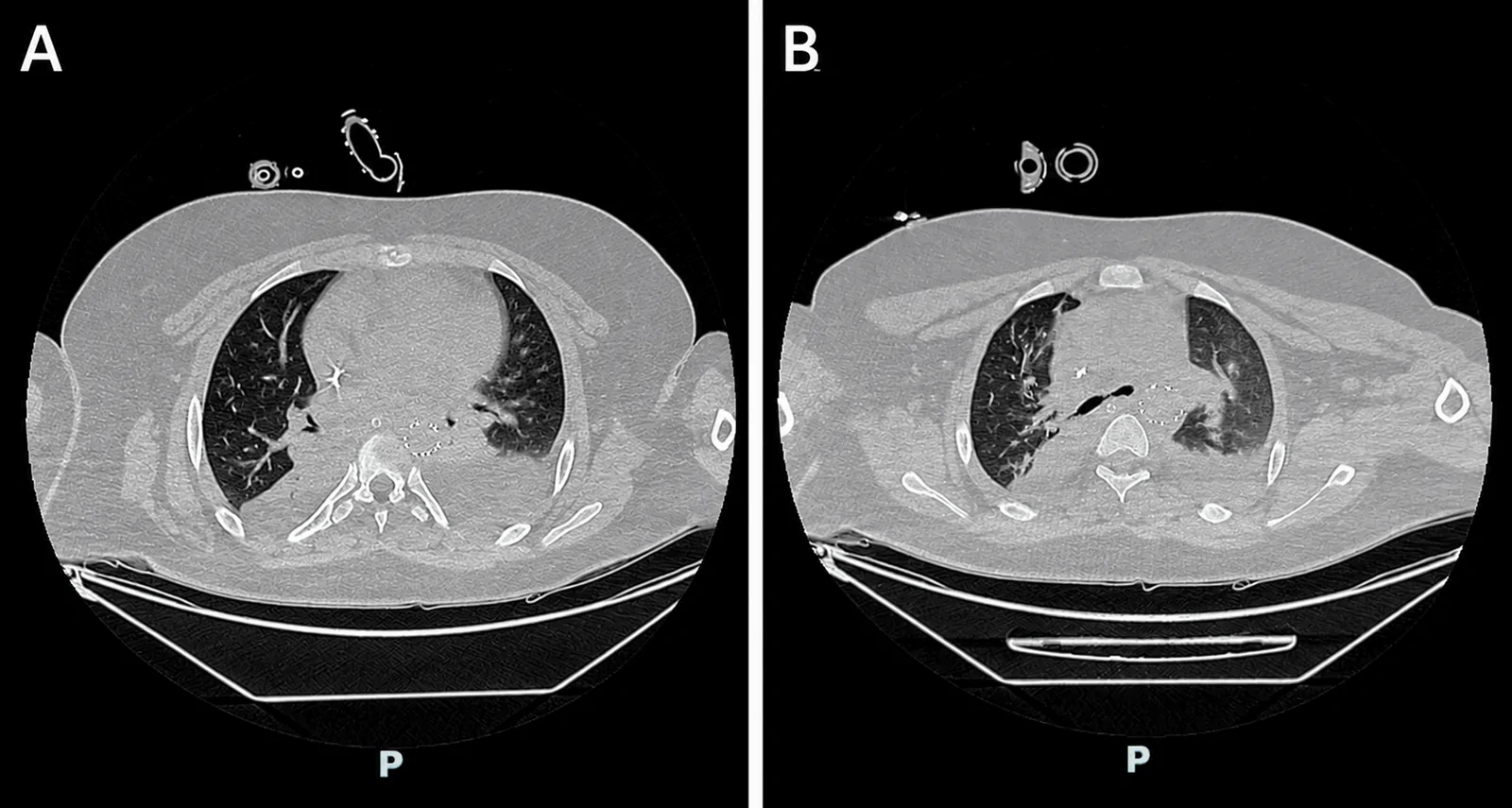
Chest computed tomography angiography during acute respiratory deterioration. (A) Axial image at the lower thoracic level demonstrates bilateral dependent consolidative-atelectatic changes, more extensive in the left lower lobe, with an associated left pleural effusion. (B) A complementary axial image at a slightly more cranial level confirms bilateral posterior pulmonary opacities and persistent left pleural fluid.

Delayed abdominal complication and further surgery

During subsequent ward and ICU care, serial vascular imaging showed a stable endograft without migration or endoleak. On hospital day 21, progressive abdominal pain and clinical deterioration prompted repeat CT, which demonstrated findings consistent with hollow-viscus perforation. Emergency laparoscopy was converted to a midline laparotomy. Terminal ileal loops were fistulized to the right lower quadrant, with ileocecal perforation and diffuse peritonitis; ileocolic resection/right hemicolectomy with ileotransverse anastomosis was performed. Postoperatively, the patient returned intubated to the ICU and was extubated the following day after successful weaning.

On day 35, after control of the abdominal and respiratory conditions, the external fixators were removed, and definitive intramedullary fixation of the left femur and tibia was completed under general anesthesia. A left pleural drain was placed during the same operative session for persistent pleural effusion. On day 44, a pelvic abscess was drained percutaneously. Rehabilitation and wound care continued thereafter.

Outcome and follow-up

The patient was discharged home on hospital day 50. At discharge, he was awake, oriented, and neurologically intact, with healing orthopedic wounds and ongoing rehabilitation needs. Follow-up CTA demonstrated a patent, correctly positioned thoracic endograft, complete exclusion of the traumatic lesion, and no endoleak. Continued vascular follow-up was arranged because of the patient’s young age and the need for long-term device surveillance. The principal clinical events and perioperative implications are summarized in Table 1. 

**Table 1:** Clinical timeline. Chronology of the major interventions and complications. ICU: intensive care unit; TEVAR: thoracic endovascular aortic repair

Hospital day	Event	Key perioperative implication
0	Emergency TEVAR	Aortic exclusion; 24 × 80-mm endograft; no endoleak
1	External fixation	Orthopedic damage control under general anesthesia
2	First extubation	Transfer from ICU after initial respiratory improvement
4-6	Reintubation and ICU care	Severe hypoxemia, bilateral consolidation, pleural effusion; subsequent successful extubation
21	Right hemicolectomy	Delayed ileocecal perforation with diffuse peritonitis
35	Definitive osteosynthesis	Femoral and tibial intramedullary fixation plus left pleural drainage
44	Pelvic abscess drainage	Percutaneous source control
50	Discharge home	Neurologically intact; endograft patent and without endoleak

## Discussion

This case illustrates three clinically relevant points. First, emergency TEVAR can provide rapid definitive control of a high-grade aortic injury in a selected adolescent, but pediatric anatomy and expected somatic growth make device selection and long-term surveillance particularly important. Contemporary pediatric data suggest increasing use of TEVAR in adolescents and favorable in-hospital outcomes in appropriately selected patients, although evidence remains predominantly retrospective [5-8]. The 2026 SVS focused update reinforces injury-grade- and physiology-based selection, avoidance of unnecessary intervention in low-grade lesions, and structured follow-up [1].

Second, the anesthetic objective changed repeatedly during hospitalization. During emergency aortic repair, priorities were prevention of rupture, preservation of organ perfusion, controlled induction, invasive monitoring, and coordination with the endovascular team. During orthopedic damage-control surgery, pulmonary contusion and recent hemorrhage shaped ventilation and fluid management. When acute hypoxemic respiratory failure developed after initial extubation, the immediate priorities became airway control, lung-protective ventilation, infection evaluation, hemodynamic support, and reassessment for pleural complications. Lung-protective ventilation remains central to the care of trauma-associated acute lung injury [10].

Third, the delayed ileocecal perforation demonstrates how occult hollow-viscus injury can declare itself after the initial life-threatening lesions have been treated. Bowel perforation is uncommon after pediatric blunt trauma, and early clinical or radiologic findings may be subtle; repeated examination and renewed imaging when symptoms evolve are therefore essential [11,12]. In this patient, prompt operative source control, short postoperative ventilation, antimicrobial therapy, and later drainage of the pelvic abscess enabled recovery while definitive orthopedic surgery was deferred until physiological conditions improved.

The central lesson is continuity rather than repetition: each anesthetic episode required a new assessment of the dominant physiological threat. A technically successful endovascular procedure did not eliminate the risks generated by pulmonary contusion, infection, bowel injury, pleural effusion, repeated surgery, immobility, and rehabilitation. The favorable outcome depended on longitudinal coordination among anesthesiologists-intensivists, vascular surgeons, orthopedic surgeons, general surgeons, radiologists, infectious diseases specialists, nurses, and rehabilitation professionals.

This is a single-patient report and cannot establish comparative effectiveness or causality. Some perioperative decisions were necessarily individualized to local resources and the patient’s changing physiology. Longer follow-up is required to assess endograft durability, aortic growth, and late reintervention risk, which remain particularly relevant after TEVAR in adolescence [13].

## Conclusions

Emergency TEVAR achieved immediate and durable exclusion of a grade III BTAI in this adolescent. Recovery, however, depended on repeated physiology-based anesthetic assessment and multidisciplinary critical care through respiratory failure, delayed bowel perforation, staged orthopedic reconstruction, pleural drainage, and intra-abdominal infection. In adolescent polytrauma, successful aortic repair should be understood as the first step in a longitudinal perioperative pathway, not the endpoint of treatment.

## References

[1] DuBose J, Kundi R, Curi MA (2026). The Society for Vascular Surgery clinical practice guideline on the management of blunt thoracic aortic injury: focused update. J Vasc Surg.

[2] Lee WA, Matsumura JS, Mitchell RS (2011). Endovascular repair of traumatic thoracic aortic injury: clinical practice guidelines of the Society for Vascular Surgery. J Vasc Surg.

[3] Fox N, Schwartz D, Salazar JH (2015). Evaluation and management of blunt traumatic aortic injury: a practice management guideline from the Eastern Association for the Surgery of Trauma. J Trauma Acute Care Surg.

[4] Starnes BW, Lundgren RS, Gunn M (2012). A new classification scheme for treating blunt aortic injury. J Vasc Surg.

[5] Grubbs H, Snyder KB, Stewart K (2025). Pediatric blunt thoracic aortic injuries: understanding the role of patient size and utilization of thoracic endovascular aortic repair. J Trauma Acute Care Surg.

[6] Malgor RD, Bilfinger TV, McCormack J, Tassiopoulos AK (2015). Outcomes of blunt thoracic aortic injury in adolescents. Ann Vasc Surg.

[7] Raulli SJ, Schneider AB, Gallaher J, Motta F, Parodi E, Farber MA, Pascarella L (2023). Trends and outcomes in management of thoracic aortic injury in children, adolescent, and mature pediatric patients using data from the National Trauma Data Bank. Ann Vasc Surg.

[8] Gunabushanam V, Mishra N, Calderin J, Glick R, Rosca M, Krishnasastry K (2010). Endovascular stenting of blunt thoracic aortic injury in an 11-year-old. J Pediatr Surg.

[9] Riley DS, Barber MS, Kienle GS (2017). CARE guidelines for case reports: explanation and elaboration document. J Clin Epidemiol.

[10] Bakowitz M, Bruns B, McCunn M (2012). Acute lung injury and the acute respiratory distress syndrome in the injured patient. Scand J Trauma Resusc Emerg Med.

[11] Brown RA, Bass DH, Rode H, Millar AJ, Cywes S (1992). Gastrointestinal tract perforation in children due to blunt abdominal trauma. Br J Surg.

[12] Cobb LM, Vinocur CD, Wagner CW, Weintraub WH (1986). Intestinal perforation due to blunt trauma in children in an era of increased nonoperative treatment. J Trauma.

[13] Jubouri M, Surkhi AO, Al-Tawil M (2024). Long-term survival and reintervention following thoracic endovascular aortic repair in blunt traumatic thoracic aortic injury: a systematic review and meta-analysis. Ann Vasc Surg.

